# Associations of dicarbonyl stress with complement activation: the CODAM study

**DOI:** 10.1007/s00125-020-05098-4

**Published:** 2020-01-28

**Authors:** Ying Xin, Elisabeth Hertle, Carla J. H. van der Kallen, Casper G. Schalkwijk, Coen D. A. Stehouwer, Marleen M. J. van Greevenbroek

**Affiliations:** 1 Department of Internal Medicine, Maastricht University Medical Centre, Universiteitssingel 50, PO Box 616, 6200 MD Maastricht, the Netherlands; 2 CARIM School for Cardiovascular Diseases, Maastricht University, Maastricht, the Netherlands; 3 Division of Endocrinology, Department of Internal Medicine, The First Affiliated Hospital of Zhengzhou University, Zhengzhou, China

**Keywords:** AGEs, Complement activation, Dicarbonyl stress, Glycation, Glyoxalase 1, Human, Methylglyoxal

## Abstract

**Aims/hypothesis:**

Reactive α-dicarbonyl compounds are major precursors of AGEs and may lead to glycation of circulating and/or cell-associated complement regulators. Glycation of complement regulatory proteins can influence their capacity to inhibit complement activation. We investigated, in a human cohort, whether greater dicarbonyl stress was associated with more complement activation.

**Methods:**

Circulating concentrations of dicarbonyl stress markers, i.e. α-dicarbonyls (methylglyoxal [MGO], glyoxal [GO] and 3-deoxyglucosone [3-DG]), and free AGEs (N^ε^-(carboxymethyl)lysine [CML], N^ε^-(carboxyethyl)lysine [CEL] and N^δ^-(5-hydro-5-methyl-4-imidazolon-2-yl)-ornithine [MG-H1]), and protein-bound AGEs (CML, CEL, pentosidine), as well as the complement activation products C3a and soluble C5b-9 (sC5b-9), were measured in 530 participants (59.5 ± 7.0 years [mean ± SD], 61% men) of the Cohort on Diabetes and Atherosclerosis Maastricht (CODAM) study. Multiple linear regression analyses were used to investigate the associations between dicarbonyl stress (standardised) and complement activation (standardised) with adjustment of potential confounders, including age, sex, lifestyle, use of medication and markers of obesity. In addition, the associations of two potentially functional polymorphisms (rs1049346, rs2736654) in the gene encoding glyoxalase 1 (GLO1), the rate-limiting detoxifying enzyme for MGO, with C3a and sC5b-9 (all standardized) were evaluated.

**Results:**

After adjustment for potential confounders, plasma concentration of the dicarbonyl GO was inversely associated with sC5b-9 (*β* −0.12 [95% CI –0.21, −0.02]) and the protein-bound AGE CEL was inversely associated with C3a (−0.17 [−0.25, −0.08]). In contrast, the protein-bound AGE pentosidine was positively associated with sC5b-9 (0.15 [0.05, 0.24]). No associations were observed for other α-dicarbonyls and other free or protein-bound AGEs with C3a or sC5b-9. Individuals with the AG and AA genotype of rs1049346 had, on average, 0.32 and 0.40 SD lower plasma concentrations of sC5b-9 than those with the GG genotype, while concentrations of C3a did not differ significantly between rs1049346 genotypes. *GLO1* rs2736654 was not associated with either C3a or sC5b-9.

**Conclusions/interpretation:**

Plasma concentrations of dicarbonyl stress markers showed distinct associations with complement activation products: some of them were inversely associated with either C3a or sC5b-9, while protein-bound pentosidine was consistently and positively associated with sC5b-9. This suggests different biological relationships of individual dicarbonyl stress markers with complement activation.

**Electronic supplementary material:**

The online version of this article (10.1007/s00125-020-05098-4) contains peer-reviewed but unedited supplementary material, which is available to authorised users.

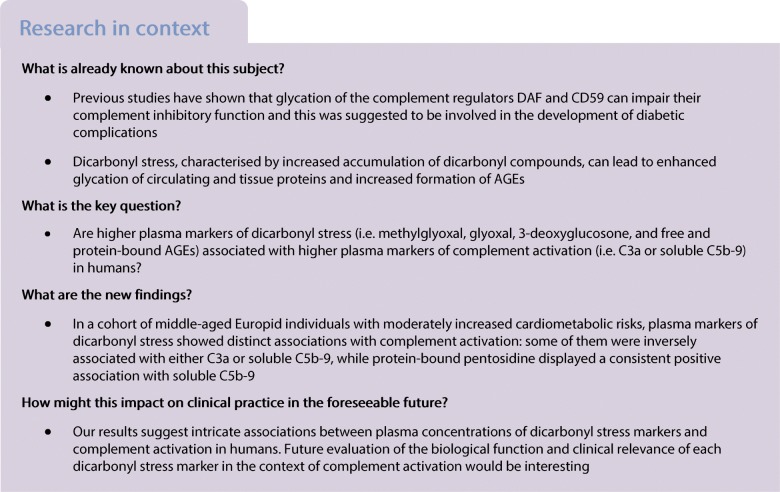



## Introduction

Chronic hyperglycaemia can induce dicarbonyl stress, which is characterised by increased presence of reactive α-dicarbonyl compounds and AGEs [1, 2]. Prolonged exposure of proteins to α-dicarbonyl compounds may change their normal function and/or their susceptibility to enzymatic degradation [3]. The complement system is part of the innate immune system and has been implicated in various cardiometabolic diseases (as reviewed in [4]). Interestingly, glycation of complement inhibitory proteins may affect complement activation [5–8], which may contribute to the development of vascular complications in people with diabetes [7].

**Fig. 1:**
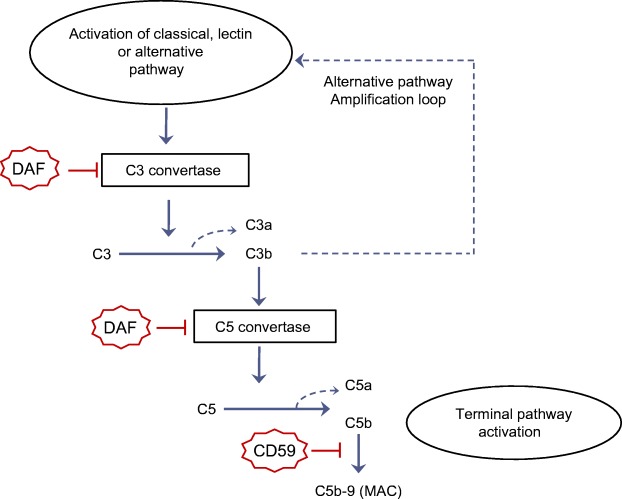
Activation and regulation of the complement system with focus on components that are most relevant for the current study. Complement activation starts with the activation of the classical, lectin, and/or the alternative pathway. Activation of these initial pathways leads to the generation of C3 convertases that cleave C3, the central complement product, into C3a and C3b. C3b produced by different complement pathways can contribute to the formation of alternative pathway C3 convertase. In this way, the alternative pathway functions as an amplification loop for all activation pathways. C3b can also lead to the generation of C5 convertase, which cleaves C5 into C5a and C5b and triggers the activation of the terminal pathway, leading to the formation of the MAC, C5b-9. DAF and CD59 are two membrane inhibitors for complement activation. DAF controls complement activation by preventing the formation and accelerating the decay of C3 and C5 convertases. CD59 mainly functions as a specific inhibitor of C5b-9 formation

During complement activation, C3 convertases that are generated can cleave the central component of the complement cascade, C3, into C3b and the anaphylatoxin C3a (as reviewed in [9], see Fig. 1). C3b can induce subsequent activation of the common terminal complement pathway resulting in the formation of C5b-9, also known as the membrane attack complex (MAC). Uncontrolled complement activation can damage host cells and/or hyperactivate inflammatory pathways [10]. To prevent this, the complement system is under strict control by circulating and cell-surface inhibitors (as reviewed in [9, 11]).

Decay accelerating factor (DAF) is a membrane-bound inhibitor of complement activation (see Fig. 1). It prevents formation of complement convertases and accelerates their decay, thereby inhibiting the generation of C3a and C3b [12]. Recently, it was shown that glycated DAF is present on erythrocytes of individuals with diabetes and this was accompanied by a less efficient control of complement activation [5]. The function of another membrane-bound inhibitor of complement activation, CD59, was also hampered by glycation [7, 8, 13]. CD59 controls the final step of terminal pathway activation (as reviewed in [9] and shown in Fig. 1). In vitro studies demonstrated that glycation of CD59 impaired its inhibitory effect on C5b-9/MAC-related lysis of human erythrocytes [13]. Also, erythrocytes of participants with diabetes were more susceptible to complement-related lysis, likely as a result of glycation-induced inactivation of CD59 [8]. Notably, in the kidney and nerves of participants with diabetes, glycated CD59 co-localised with C5b-9, and presence of glycated, less functional CD59 was considered to be the cause of increased C5b-9 deposition [7]. Glycated CD59 was also higher in the circulation of diabetic than of healthy individuals [6], and was detected in the urine of participants with diabetes [13]. Glycation of other complement components, such as C3 [3, 14–17] and factor B [18] has also been reported, but biological effects of glycation on their function, if any, have not yet been reported.

Taken together, existing data suggest that dicarbonyl stress and subsequent glycation of complement inhibitors may impair their regulation on complement activation. In this study we investigated to what extent dicarbonyl stress, as reflected by the presence of dicarbonyls and AGEs in the circulation, is related to complement activation, as represented by the plasma concentrations of two complement activation products, i.e. C3a and soluble (s)C5b-9.

## Methods

### Study population

The present study used the data of the Cohort on Diabetes and Atherosclerosis Maastricht (CODAM) study. The CODAM study was designed to investigate possible contributors to the natural development of type 2 diabetes and cardiovascular disease [19]. Briefly, participants from a large population-based observational study were invited for screening for the CODAM study if they were Europid, >40 years old and had one or more of the following characteristics: BMI >25 kg/m^2^; positive family history of type 2 diabetes; postprandial glucose level >6.0 mmol/l; history of gestational diabetes and/or glucosuria; and use of antihypertensive medication. In total, 574 individuals were recruited. This study was approved by the medical ethics committee of Maastricht University. All participants gave written informed consent.

Individuals with missing data on the main variables, and/or important covariates, were excluded (*n* = 44), leaving 530 individuals with complete data for the main analyses. Participants were asked to stop their lipid-modifying medication 14 days, and any other medication one day, prior to the measurements.

### Measurements of plasma α-dicarbonyls and AGEs

Peripheral blood samples were obtained after overnight fasting. To collect plasma, blood samples were kept in pre-cooled EDTA or citrate tubes on ice until the centrifugation at 1950 *g* for 15 min at 4°C (within 3 h). For serum, blood samples were allowed to clot at room temperature for 45 min and were centrifuged at 1950 *g* for 15 min to collect the serum. Plasma and serum aliquots were stored at −80°C until use. α-dicarbonyls (methylglyoxal [MGO], glyoxal [GO], 3-deoxyglucosone [3-DG]), free AGEs (N^ε^-(carboxymethyl)lysine [CML], N^ε^-(carboxyethyl)lysine [CEL], N^δ^-(5-hydro-5-methyl-4-imidazolon-2-yl)-ornithine [MG-H1]), and protein-bound CML and CEL were measured in EDTA plasma by ultra HPLC-tandem MS (Waters, Milford, MA, USA). Protein-bound pentosidine was measured in EDTA plasma by HPLC (Alltech/Grace, Breda, the Netherlands) as previously described [20, 21]. The interassay variations were 4.3%, 5.1%, 2.2%, 7.1%, 6.4%, 5.1%, 7.0%, 9.7% and 3.1% for MGO, GO, 3-DG, free CML, free CEL, free MG-H1, protein-bound CML, protein-bound CEL and protein-bound pentosidine, respectively.

### Genotyping of *GLO1* polymorphisms

SNP rs2736654 [22, 23] and rs1049346 [24], two functional polymorphisms in the gene encoding glyoxalase 1 (GLO1), were genotyped in blood samples by the ABI PRISM 7900HT sequence detection system (Applied Biosystems, Foster City, California, USA), as previously described [25].

### Measurements of complement factors

C3a was measured in EDTA plasma and sC5b-9 was measured in citrate plasma by ELISA (MicroVue C3a plus EIA kit, MicroVue sC5b-9 EIA kit, Quidel, San Diego, CA, USA) as previously described [26, 27]. The interassay variations were 6.6% and 11.8% for C3a and sC5b-9. C3 was determined in serum by auto-analyser (Hitachi 912) using Roche kit assays (Roche Diagnostics Nederland, Almere, the Netherlands), and in EDTA plasma by IMMAGE immunochemistry system C3 assay (Beckman-Coulter, Brea, CA, USA). The interassay variations for these measurements was 2.1% and 7.0%, respectively. The mean value of these two measurements (after calibration) was used for further analyses.

### Measurements of other covariates

Other covariates were obtained as described before. Briefly, BMI (kg/m^2^) and waist circumference (cm) were measured at the research facility [19]. Information on medication use (glucose-lowering, lipid-modifying and/or antihypertensive, each yes/no), lifestyle factors (smoking status [current or previous tobacco smoking, yes/no], physical activity [METs/week], total energy intake [kJ/day], and alcohol consumption [g/day]) were obtained via questionnaires [19]. Normal glucose metabolism (NGM) (yes/no), impaired glucose metabolism (IGM) (yes/no) and type 2 diabetes (yes/no) were defined according to the 1999 WHO criteria based on OGTT data [28]. Concentrations of triacylglycerol, total cholesterol, HDL-cholesterol and creatinine (to estimate eGFR [29]) were measured in fasting blood samples [21].

### Statistical analyses

General characteristics of the study population were compared between individuals with lower or higher than median concentrations of C3a and sC5b-9 by using independent sample *t* tests, Mann–Whitney *U* tests or Pearson *χ*^2^ tests. Variables with skewed distribution (i.e. free and protein-bound CML and CEL, free MG-H1, protein-bound pentosidine, C3a, fasting plasma glucose, alcohol intake and triacylglycerol) were log_2_-transformed prior to further analyses. All analyses were performed using IBM SPSS statistics version 25, and a two-tailed *p* value <0.10 (for interaction terms) or <0.05 (all other analyses) was considered significant. Given the large number of statistical analyses that were conducted, a false discovery rate (FDR) adjusted *p* value of <0.05 was additionally calculated for the main results (Tables 2 and 3).

#### Main analyses

Multiple linear regression analyses were used to investigate the associations of markers of dicarbonyl stress (α-dicarbonyls and AGEs, main independent variables), with markers of complement activation (C3a and sC5b-9, main outcomes). Standardised values were calculated ([individual observed values − population mean]/SD of the population) for the main independent and dependent variables to allow direct comparison of the effect sizes. All analyses were adjusted for age and sex (model 1), then additionally for lifestyle factors and use of medication (model 2), and for waist circumference (model 3, fully adjusted model) to control for potential confounding.

Plasma lipids (triacylglycerol, total cholesterol, HDL-cholesterol), renal function (eGFR), and C3 concentration were added separately to model 3. Because of the intricate biological relationships of these covariates with both dicarbonyl stress and complement, these additional models may to some extent be overadjusted. In addition, since the level of protein glycation will differ between individuals with and without diabetes or IGM, glucose metabolism status (IGM and type 2 diabetes, yes/no as dummy variables) was added to model 3 and the effect of prevalent diabetes (type 2 diabetes, yes/no) on the associations of interest was evaluated using interaction analyses.

#### Sensitivity analyses

Some disease conditions may affect plasma concentrations of complement proteins. Therefore, the main analyses were repeated after excluding participants with: (1) acute or chronic infections (C-reactive protein >95.2 nmol/l); (2) a (suspected) history of autoimmune disease, defined as self-reported current chronic joint inflammation/rheumatoid arthritis or a severe intestinal disorder that lasted for the past 3 months or longer; (3) a self-reported current malignant condition or cancer; or (4) self-reported liver disease. These sensitivity analyses were performed in 494, 459, 508 and 524 individuals for the four conditions, respectively.

#### Additional analyses

We investigated the associations of two common functional polymorphisms in *GLO1*, rs2736654 and rs1049346, with C3a and sC5b-9. GLO1 is the rate-limiting detoxifying enzyme for MGO, the most reactive dicarbonyl in the formation of AGEs. Adjustment for confounders was done as for the main analyses. These additional analyses were performed in 504 individuals because information on *GLO1* polymorphisms was missing in 26 participants.

## Results

### General characteristics of the study population

General characteristics of the participants are shown in Table 1. Individuals with higher C3a concentrations were more often women, had higher measures of adiposity, and lower daily alcohol consumption and physical activity compared with those with lower C3a concentrations. They had lower plasma concentrations of protein-bound CEL and pentosidine. Individuals with higher sC5b-9 concentrations did not show obvious differences in general characteristics, but had lower GO concentrations compared with those with lower sC5b-9 concentrations. Individuals with higher plasma C3a generally had higher sC5b-9, and vice versa.

**Table 1:** Characteristics of the total population and according to lower and higher concentrations of C3a and sC5b-9

Variable	Total study population ^a^	Median of C3a concentration			Median of sC5b-9 concentration		
		<Median	>Median	*p* value	<Median	>Median	*p* value
Age (years)	59.5 ± 7.0	59.6 ± 6.6	59.3 ± 7.4	0.668	59.5 ± 6.9	59.4 ± 7.1	0.949
Sex (% men)	61	67	55	0.004	65	57	0.075
BMI (kg/m^2^)	28.6 ± 4.4	27.8 ± 3.9	29.3 ± 4.7	<0.001	28.3 ± 4.1	28.8 ± 4.6	0.166
Waist circumference (cm)	99.3 ± 12.0	97.8 ± 10.6	100.8 ± 13.2	0.004	98.9 ± 11.1	99.7 ± 12.9	0.435
Fasting plasma glucose (mmol/l)	5.6 (5.2–6.5)	5.7 (5.3–6.4)	5.5 (5.1–6.5)	0.324	5.7 (5.2–6.5)	5.6 (5.2–6.4)	0.288
HbA_1c_ (mmol/mol)	41.9 ± 9.1	41.9 ± 9.9	41.8 ± 8.3	0.972	41.9 ± 10.3	41.9 ± 7.8	0.997
HbA_1c_ (%)	6.0 ± 0.8	6.0 ± 0.9	6.0 ± 0.8	–	6.0 ± 0.9	6.0 ± 0.7	–
Glucose metabolism status							
NGM/IGM/T2DM (%)	52/22/26	53/20/26	51/24/26	0.638	52/22/26	52/22/26	0.980
Current or previous smokers (%)	23	20	25	0.179	25	21	0.352
Alcohol intake (g/day)	8.6 (1.3–22.6)	10.1 (2.3–25.7)	6.6 (0.7–18.1)	0.005	8.6 (1.3–21.2)	8.6 (1.3–24.6)	0.686
Energy intake (10^3^ kJ/day)	9.3 ± 2.8	9.5 ± 2.7	9.1 ± 2.9	0.088	9.3 ± 2.8	9.2 ± 2.8	0.545
Physical activity (10^3^ METs/week)	6.6 ± 4.1	7.0 ± 4.4	6.3 ± 3.7	0.038	6.8 ± 4.1	6.5 ± 4.1	0.461
Glucose-lowering medication (%)	14	15	13	0.447	15	13	0.447
Lipid-modifying medication (%)	19	17	22	0.227	19	20	0.742
Antihypertensive medication (%)	39	36	42	0.109	36	42	0.109
Total cholesterol (mmol/l)	5.2 ± 1.0	5.2 ± 0.9	5.2 ± 1.0	0.904	5.2 ± 1.0	5.2 ± 1.0	0.821
Total triacylglycerol (mmol/l)	1.4 (1.0–2.0)	1.4 (1.0–2.0)	1.4 (1.0–1.9)	0.434	1.4 (1.0–2.0)	1.4 (0.9–1.9)	0.099
HDL-cholesterol (mmol/l)	1.2 ± 0.3	1.2 ± 0.3	1.2 ± 0.3	0.318	1.2 ± 0.3	1.2 ± 0.4	0.691
eGFR (ml min^−1^ [1.73 m]^2^)	91.3 ± 18.3	91.9 ± 17.9	90.7 ± 18.8	0.452	91.9 ± 17.7	90.7 ± 18.9	0.457
C3a (μg/l)	59.2 (50.1–72.7)	50.1 (44.1–54.3)	72.7 (65.2–85.7)	–	56.6 (48.0–70.0)	62.8 (51.8–75.9)	<0.001
sC5b-9 (μg/l)	113.0 ± 32.9	107.6 ± 30.6	118.4 ± 34.3	<0.001	87.1 ± 15.5	138.8 ± 24.2	–
C3 (g/l)	1.0 ± 0.2	1.0 ± 0.1	1.1 ± 0.2	<0.001	0.99 ± 0.15	1.04 ± 0.16	<0.001
α-dicarbonyls							
MGO (nmol/l)	367.4 ± 77.7	368.8 ± 77.8	366.0 ± 77.8	0.680	369.2 ± 72.4	365.5 ± 82.9	0.579
GO (nmol/l)	1140.5 ± 359.3	1164.8 ± 365.1	1116.3 ± 352.4	0.120	1182.4 ± 354.3	1098.6 ± 360.0	0.007
3-DG (nmol/l)	1283.3 ± 388.7	1297.3 ± 417.5	1269.3 ± 357.9	0.408	1284.3 ± 379.3	1282.3 ± 398.6	0.952
Free AGEs							
CML (nmol/l)	78.3 (60.5–98.1)	78.4 (60.4–93.3)	78.2 (61.4–101.3)	0.204	81.8 (63.5–98.4)	76.1 (58.2–97.9)	0.505
CEL (nmol/l)	45.2 (36.5–58.2)	43.7 (36.7–58.7)	45.6 (35.7–57.0)	0.788	45.9 (38.1–59.9)	43.4 (35.5–56.0)	0.083
MG-H1 (nmol/l)	122.3 (86.3–174.7)	118.1 (85.6–170.0)	125.9 (86.6–178.1)	0.319	125.1 (88.6–176.6)	119.6 (82.8–172.6)	0.474
Protein-bound AGEs							
CML (nmol/mmol lysine)	34.4 (29.5–40.8)	34.6 (30.5–41.4)	34.1 (28.9–40.6)	0.073	34.4 (29.7–41.5)	34.5 (28.9–40.3)	0.450
CEL (nmol/mmol lysine)	23.1 (18.9–29.0)	24.4 (20.0–30.3)	21.9 (17.8–27.0)	<0.001	23.7 (19.1–29.5)	23.0 (18.4–28.2)	0.076
Pentosidine (nmol/mmol lysine)	0.43 (0.36–0.53)	0.45 (0.38–0.54)	0.42 (0.36–0.51)	0.026	0.43 (0.37–0.50)	0.43 (0.36–0.55)	0.116

Variables are presented as mean ± SD, percentages, or median (interquartile range)

^a^Data from 530 participants were included in the main analyses. Data for BMI were available for *n* = 529; for HbA_1c_ were available for *n* = 505; for fasting plasma glucose were available for *n* = 529

T2DM, type 2 diabetes mellitus

### Associations of plasma α-dicarbonyls with complement activation

No associations were observed for α-dicarbonyls with C3a, or for MGO and 3-DG with sC5b-9 (Table 2). GO was inversely associated with sC5b-9 after the adjustment for potential confounders. This association was significant at the nominal *p* value (Table 2, model 3, sC5b-9, *β* = −0.12 [95% CI –0.21, −0.02]) but did not reach an FDR *q* value <0.05. Associations of α-dicarbonyls with C3a and sC5b-9 were virtually unchanged after additional adjustment for plasma lipids, glucose metabolism status, renal function or plasma C3 (electronic supplementary material [ESM] Table 1). The inverse association of GO with sC5b-9 was attenuated and became non-significant after the adjustment for C3 (Table S1, *β* = −0.08 [−0.17, 0.01]). When we evaluated if the association of α-dicarbonyls with complement activation differed between individuals with and without type 2 diabetes, significant interactions were only observed for MGO (ESM Table 2). In subsequent stratified analyses, there were positive associations, although non-significant, of MGO with both C3a and sC5b-9 in type 2 diabetes (C3a, *β* = 0.13 [−0.01, 0.28]; sC5b-9, *β* = 0.13 [−0.05, 0.31]; *n* = 138), but not in those without diabetes (C3a, *β* = −0.04 [−0.15, 0.07]; sC5b-9, *β* = −0.05 [−0.15, 0.06]; *n* = 392).

**Table 2:** Associations of plasma α-dicarbonyls, free and protein-bound AGEs with C3a and sC5b-9 (*n* = 530)

Variable	Model	C3a (SD)			sC5b-9 (SD)		
		*β*	95% CI	*p* value	*β*	95% CI	*p* value
α-dicarbonyls							
MGO (SD)	1	0.01	(−0.07, 0.10)	0.773	0.01	(−0.08, 0.10)	0.825
	2	0.02	(−0.07, 0.11)	0.619	0.02	(−0.07, 0.11)	0.711
	3	0.01	(−0.08, 0.10)	0.812	0.02	(−0.07, 0.10)	0.745
GO (SD)	1	−0.08	(−0.16, 0.01)	0.074	−0.13	(−0.21, −0.04)	0.004 *
	2	−0.06	(−0.15, 0.03)	0.162	−0.12	(−0.21, −0.03)	0.012
	3	−0.06	(−0.14, 0.03)	0.218	−0.12	(−0.21, −0.02)	0.013
3-DG (SD)	1	0.02	(−0.07, 0.11)	0.659	0.04	(−0.05, 0.13)	0.372
	2	0.04	(−0.06, 0.15)	0.428	0.08	(−0.03, 0.19)	0.141
	3	0.00	(−0.10, 0.11)	0.955	0.08	(−0.03, 0.18)	0.174
Free AGEs							
CML (SD)	1	0.03	(−0.06, 0.12)	0.458	−0.02	(−0.11, 0.07)	0.637
	2	0.01	(−0.07, 0.10)	0.792	−0.02	(−0.11, 0.07)	0.648
	3	0.01	(−0.08, 0.10)	0.856	−0.02	(−0.11, 0.07)	0.638
CEL (SD)	1	−0.02	(−0.11, 0.06)	0.610	−0.07	(−0.15, 0.02)	0.139
	2	−0.04	(−0.13, 0.05)	0.394	−0.07	(−0.15, 0.02)	0.146
	3	−0.05	(−0.14, 0.04)	0.262	−0.07	(−0.16, 0.02)	0.133
MG-H1 (SD)	1	−0.03	(−0.12, 0.06)	0.508	−0.04	(−0.13, 0.05)	0.356
	2	−0.05	(−0.14, 0.04)	0.236	−0.03	(−0.13, 0.06)	0.467
	3	−0.05	(−0.13, 0.04)	0.311	−0.03	(−0.12, 0.06)	0.488
Protein-bound AGEs							
CML (SD)	1	−0.14	(−0.23, −0.06)	0.001*	−0.07	(−0.15, 0.02)	0.136
	2	−0.13	(−0.22, −0.05)	0.002*	−0.05	(−0.14, 0.04)	0.243
	3	−0.08	(−0.17, −0.01)	0.088	−0.05	(−0.15, 0.05)	0.334
CEL (SD)	1	−0.16	(−0.24, −0.08)	<0.001*	−0.07	(−0.16, 0.01)	0.087
	2	−0.17	(−0.25, −0.08)	<0.001*	−0.08	(−0.17, 0.00)	0.057
	3	−0.17	(−0.25, −0.08)	<0.001*	−0.08	(−0.17, 0.00)	0.058
Pentosidine (SD)	1	−0.11	(−0.20, −0.03)	0.010 *	0.10	(0.02, 0.19)	0.020
	2	−0.11	(−0.19, −0.02)	0.017	0.12	(0.03, 0.21)	0.007 *
	3	−0.05	(−0.14, −0.04)	0.246	0.15	(0.05, 0.24)	0.002*

Concentrations of α-dicarbonyls, AGEs, C3a and sC5b-9 were standardised. Concentrations of AGEs and C3a were log_2_-transformed prior to standardisation

Model 1: adjusted for age and sex. Model 2: model 1 + lifestyle (smoking status, alcohol consumption, physical activity and energy intake) and medication use (glucose-lowering, lipid-modifying and/or antihypertensive). Model 3: model 2 + waist circumference

*FDR-adjusted *q* value <0.05

### Associations of plasma free AGEs with complement activation

Free AGEs were not associated with either C3a or sC5b-9 (Table 2, ESM Table 1). Diabetes status influenced the associations of free CML and CEL with sC5b-9, but not the associations of other free AGEs with C3a or sC5b-9 (ESM Table 2). In subsequent stratified analyses, significant inverse associations were observed for free CML and CEL with sC5b-9 in individuals with diabetes (free CML, *β* = −0.21 [95% CI –0.41, −0.01]; free CEL, *β* = −0.29 [−0.47, −0.10]), but not in those without diabetes (free CML, *β* = 0.04 [−0.06, 0.14]; free CEL, *β* = 0.01 [−0.09, 0.11]).

### Associations of plasma protein-bound AGEs with complement activation

In the age- and sex-adjusted regression models, protein-bound CML, CEL and pentosidine were inversely and significantly associated with C3a, which remained significant after further adjustment for lifestyle and medication use (Table 2, model 2, protein-bound CML, *β* = −0.13 [95% CI –0.22, −0.05]; protein-bound CEL, *β* = −0.17 [−0.25, −0.08]; protein-bound pentosidine, *β* = −0.11 [−0.19, −0.02]). After additional adjustment for waist circumference, the associations for protein-bound CML and pentosidine were attenuated and became non-significant, while the association for protein-bound CEL remained significant (Table 2, model 3, protein-bound CML, *β* = −0.08 [−0.17, 0.01]; protein-bound pentosidine, *β* = −0.05 [−0.14, 0.04]; protein-bound CEL, *β* = −0.17 [−0.25, −0.08]). In contrast, after adjustment for potential confounders, a positive association was observed for protein-bound pentosidine with sC5b-9 (Table 2, model 3, *β* = 0.15 [0.05, 0.24]). No associations were observed for protein-bound CML or CEL with sC5b-9 (Table 2).

These associations were mostly unchanged after additional adjustment for plasma lipids, glucose metabolism status, renal function or C3 (ESM Table 1). Only the inverse association of protein-bound CEL with sC5b-9 became slightly stronger and was borderline significant after additional adjustment for eGFR (*β* = −0.09 [95% CI –0.17, −0.00]) and for C3 (*β* = −0.09 [−0.17, −0.00]). In addition, the associations of protein-bound CEL with C3a, and of protein-bound CML and CEL with sC5b-9, were different in individuals with and without diabetes (ESM Table 2). Protein-bound CEL was inversely associated with C3a in individuals without diabetes (*β* = −0.23 [−0.33, −0.12]), and not in those with diabetes (*β* = −0.05 [−0.19, 0.08]). In contrast, protein-bound CML and CEL were inversely and significantly associated with sC5b-9 in diabetes (protein-bound CML, *β* = −0.24 [−0.42, −0.06], protein-bound CEL, *β* = −0.21 [−0.38, −0.05]), but not in individuals without diabetes (protein-bound CML, *β* = 0.05 [−0.07, 0.16], protein-bound CEL, *β* = −0.02 [−0.12, 0.08]).

### Sensitivity analyses

Most associations were not materially changed after excluding participants with acute or chronic infections, with a (suspected) history of autoimmune disease, with a self-reported current malignant condition/cancer, or with a self-reported liver disease (ESM Table 3). The inverse association of GO with sC5b-9 was attenuated and became non-significant when participants with acute or chronic infections were excluded (*β* = −0.08 [95% CI –0.18, 0.01]).

### Associations of functional *GLO1* polymorphism with C3a and sC5b-9

Genotyping success for *GLO1* rs2736654 and rs1049346 was 97.2% and 97.0%, respectively. The two *GLO1* polymorphisms were not significantly associated with C3a and no significant associations were observed for rs2736654 with sC5b-9 (Table 3). In contrast, significant inverse associations were observed for rs1049346 with sC5b-9 (Table 3, model 3, AG *β* = −0.32 [95% CI –0.54, −0.12]; AA *β* = −0.40 [−0.65, −0.15]). This implies that, compared with those with the GG genotype, individuals with the AG genotype had, on average, 0.32 SD lower plasma sC5b-9, and those with the AA genotype had, on average, 0.40 SD lower sC5b-9. The associations of the two *GLO1* polymorphisms with MGO, free and protein-bound AGEs were also evaluated, and almost no associations were observed, except an inverse association of the GT genotype of rs2736654 with MGO (ESM Table 4, model 3, *β* = −0.21, *p* = 0.036).

**Table 3:** Associations of *GLO1* polymorphisms with C3a and sC5b-9 (*n* = 504)

	*n* (%)	Model	C3a (SD)			sC5b-9 (SD)		
			*β*	95% CI	*p* value	*β*	95% CI	*p* value
rs2736654								
TT	155 (31%)	reference	–	–	–	–	–	–
GT	259 (51%)	1	0.00	(−0.20, 0.20)	0.998	−0.01	(−0.21, 0.19)	0.891
		2	−0.02	(−0.22, 0.17)	0.812	−0.03	(−0.23, 0.17)	0.758
		3	−0.04	(−0.24, 0.15)	0.676	−0.03	(−0.23, 0.17)	0.750
GG	90 (18%)	1	−0.08	(−0.34, 0.18)	0.559	−0.20	(−0.46, 0.06)	0.127
		2	−0.10	(−0.36, 0.16)	0.430	−0.22	(−0.48, 0.04)	0.102
		3	−0.08	(−0.34, 0.18)	0.533	−0.22	(−0.48, 0.05)	0.105
rs1049346								
GG	116 (23%)	reference	–	–	–	–	–	–
AG	260 (52%)	1	−0.06	(−0.28, 0.16)	0.595	−0.30	(−0.51, −0.08)	0.008 *
		2	−0.08	(−0.29, 0.14)	0.494	−0.32	(−0.54, −0.10)	0.004*
		3	−0.08	(−0.29, 0.14)	0.493	−0.32	(−0.54, −0.10)	0.004*
AA	128 (25%)	1	−0.13	(−0.38, 0.12)	0.297	−0.37	(−0.62, −0.12)	0.004 *
		2	−0.17	(−0.42, 0.08)	0.193	−0.40	(−0.65, −0.15)	0.002*
		3	−0.16	(−0.40, 0.09)	0.217	−0.40	(−0.65, −0.15)	0.002 *

The reference categories are the genotypes that were reported to be associated with the highest GLO1 activity (see references [22–24])

Concentrations of C3a and sC5b-9 were standardised. Concentrations of C3a were log_2_-transformed prior to standardisation

Model 1: adjusted for age and sex. Model 2: model 1 + lifestyle (smoking status, alcohol consumption, physical activity and energy intake) and medication use (glucose-lowering, lipid-modifying and/or antihypertensive). Model 3: model 2 + waist circumference

*FDR-adjusted *q* value <0.05

## Discussion

We hypothesised that greater dicarbonyl stress, as reflected by higher plasma concentrations of reactive α-dicarbonyl compounds and related AGEs, leads to glycation of complement inhibitors, resulting in impaired control of complement activation and hence increased formation of complement activation products. Our study has several main findings. First, in the whole study population, GO was inversely associated with sC5b-9 while no associations were observed for other α-dicarbonyls with C3a or sC5b-9. Second, in the whole study population, free AGEs were not associated with C3a or sC5b-9, although free CML and CEL were inversely associated with sC5b-9 in individuals with type 2 diabetes. Third, protein-bound CEL, and to a lesser extent CML and pentosidine, were inversely associated with C3a; protein-bound CML and CEL were not associated with sC5b-9 in the whole population but were inversely associated with sC5b-9 in individuals with type 2 diabetes. In contrast, protein-bound pentosidine was positively and independently associated with sC5b-9, regardless of the diabetes status. Finally, C3a did not differ between genotypes of the two *GLO1* polymorphisms. sC5b-9 did not differ between the genotypes of rs2736654, while individuals with the AA and AG genotypes of rs1049346 had lower sC5b-9 concentrations than those with GG genotype.

The inverse association between GO and sC5b-9 was an unexpected observation in the light of our pre-specified hypothesis. Notably, this relationship was attenuated and no longer significant after adjustment for C3, and after exclusion of participants with acute or chronic infections. This attenuation suggests that this association was, at least partly, due to an ongoing inflammatory process that is somehow related to lower plasma GO concentration and/or that plasma GO may have anti-inflammatory properties. GO is generally considered a proinflammatory compound, although information on its association with inflammation in humans is scarce. One experimental study showed that exogenous GO can induce inflammatory injury in human vascular endothelial cells [30]. We previously showed that plasma sC5b-9 is positively associated with low-grade inflammation [27]. Our current observation might thus be explained by a mechanism similar to what was previously reported for CML [31]: plasma GO may be transported to tissue or interstitial fluid where it can contribute to complement activation and the inflammation process. Therefore, the decrease in plasma GO, as a result of increased uptake into tissue, may contribute to local complement activation and related inflammation that is subsequently reflected in the plasma compartment.

The inverse association of protein-bound CML with C3a was largely explained by obesity. We and others previously showed that plasma protein-bound CML was inversely associated with central obesity, at least partly because it was trapped by visceral adipose tissue via the receptor for AGEs (RAGE) [31–33] which, in combination with the positive association between plasma C3a and obesity [34, 35], may explain the current inverse association. Serum pentosidine was also inversely correlated with BMI in one recent human study [36]. Given that pentosidine can also bind to RAGE [37], a similar obesity-dependent inverse association may underlie the inverse association between pentosidine and C3a, which was confounded by central obesity.

The inverse association between protein-bound CEL and C3a was independent of all confounders included in our analyses. Interestingly, this association was only present in individuals without diabetes and not in those with diabetes. Thus, this inverse association might be diminished in hyperglycaemia/diabetes. In contrast, in diabetes, strong inverse associations of free and protein-bound CML and CEL with sC5b-9 were seen. Although the underlying mechanism of these unexpected diabetes-specific associations is not clear yet, this may suggest that the associations of these dicarbonyls and AGEs with C3a and sC5b-9 occurs via different routes.

In line with our hypothesis, we observed a consistent positive association between protein-bound pentosidine and sC5b-9. This association was independent of possible confounders, was not influenced by diabetes, and remained significant in all sensitivity analyses. A possible explanation is that protein-bound pentosidine may, better than the other AGEs in our analyses, reflect the tissue-AGE content. In that line, we previously reported that skin autofluorescence (a non-invasive measurement of skin AGE accumulation) and plasma protein-bound pentosidine were both positively associated with aortic stiffening, while protein-bound CML and CEL were not [38]. Plasma pentosidine also correlated with skin autofluorescence in Japanese haemodialysis patients [39]. Yet another small case–control study also reported a non-significant positive association between serum protein-bound pentosidine and pentosidine levels in skin biopsies [40]. The positive association between plasma protein-bound pentosidine and sC5b-9 that we observed may thus reflect a potential positive relationship between protein glycation in tissues and complement activation.

Overall, our observations reveal distinct associations of plasma dicarbonyl compounds and AGEs with C3a and sC5b-9. This suggests that, instead of being a general reflection of dicarbonyl stress, these plasma markers may each reflect distinct pathophysiological processes. Indeed, different biological effects for these compounds have been reported. For instance, MGO and GO may trigger distinct intracellular signals involved in various cellular functions in cultured endothelial cells, because of differences in their chemical structures [41]. The possibility of different contributions for AGEs in the development of vascular disease was also reported [38]. For instance, via binding to RAGE, AGEs like CML can activate inflammatory pathways (as reviewed in [42]), while cross-linking AGEs such as pentosidine may act via formation of cross-links between extracellular matrix proteins [43, 44]. In addition, the different associations we observe for C3a and sC5b-9 could be explained by the fact that complement regulators differ in their exclusivity for generation of C3a and C5b-9. CD59 is the dedicated regulator of the formation of C5b-9/MAC, while there are several other complement regulators that could compensate the inhibition of DAF on C3 activation.

Plasma dicarbonyls produce a snapshot of current dicarbonyl stress while AGEs rather represent dicarbonyl stress over the last days or weeks. In our additional analyses, we included a measure that may reflect lifelong differences in dicarbonyl stress, i.e. potentially functional variants of the MGO-detoxifying enzyme GLO1. Some human studies on *GLO1* rs2736654 reported that individuals with AA (i.e. TT) genotype have the best GLO1 function/activity [22, 23]. This implies that individuals with the AC (i.e. GT) or CC (i.e. GG) genotype may have been exposed to lifelong greater dicarbonyl stress which, according to our hypothesis, would lead to more complement activation. rs2736654 was not associated with C3a concentrations. Moreover, individuals carrying the genotype with the lowest predicted GLO1 activity (i.e. the GG genotype) had lower sC5b-9 concentrations, although non-significant. rs1049346 was also not associated with C3a concentration, whereas individuals with AG or AA genotypes of rs1049346, which were reported to have lower GLO1 enzyme activity [24], had significantly lower sC5b-9 concentrations. Critical re-evaluation of the available literature shows that the claims on functionality of these polymorphisms were partly based on in vitro data and partly inconsistent [22–24, 45, 46]. This hampers the interpretation of the relatively strong association of rs1049346 with C5b-9.

The main strength of our study is the availability of plasma concentrations of α-dicarbonyls, free AGEs and protein-bound AGEs, as well as complement activation products, within one well-phenotyped cohort. The detailed phenotyping of the study population provided us with the opportunity to thoroughly evaluate the effects of potential confounders and perform relevant sensitivity analyses. Our study also has several limitations. Most importantly, despite the information we have on the overall concentrations of dicarbonyl compounds and AGEs for each participant, we do not have information on the extent to which their individual complement regulators were actually carbonylated and/or glycated. Other limitations include the cross-sectional design, which prohibits conclusions on causality. We aimed to mitigate this limitation by including the functional *GLO1* polymorphisms, which theoretically represent a lifelong exposure to greater dicarbonyl stress, at least to MGO. Further exploration on the effects of *GLO1* polymorphisms on GLO1 function and dicarbonyl stress is needed, since we cannot exclude the possibility that the relatively small sample size of our cohort may have limited the power of the present study to detect associations of *GLO1* polymorphisms with complement activation. Moreover, in the analyses stratified for presence of diabetes, the smaller sample size in subgroups may have further decreased the statistical power to identify relevant relationships. Finally, our participants are middle-aged to older Europid individuals characterised by a moderately increased risk of cardiovascular disease. This selection of the study population limits the generalisability of present findings. Therefore, it is important that these evaluations will, in due time, be confirmed in a population-based cohort and among other ethnicities.

In conclusion, plasma concentrations of dicarbonyl stress markers displayed various associations with complement activation. This suggests different biological effects of the individual plasma α-dicarbonyls and AGEs, potentially with different clinical relevance. Our most striking findings are the unexpected inverse associations between GO and sC5b-9, between CEL and C3a. Moreover, protein-bound and free CML and CEL were inversely associated with sC5b-9, but only in diabetes. In addition, a genotype that may represent less GLO1 activity (hence greater dicarbonyl stress) was associated with less activation of the terminal pathway. Protein-bound pentosidine, on the other hand, was positively and significantly associated with sC5b-9, which was in line with our pre-specified hypothesis. Taken together, these data illustrate that the complex underlying physiological processes of these circulating markers should be taken into account in future work. The consistent associations observed for plasma pentosidine indicate that it may have priority as a marker for tissue AGEs. Moreover, the observed inverse associations between AGEs and complement activation, as well as the potential interactions with diabetes, need to be confirmed and extended in a larger study population.

## Electronic supplementary material

ESM Tables (PDF 391 kb)

## Data Availability

The datasets analysed during the current study are available from the corresponding author on reasonable request.

## Abbreviations

CEL: N^ε^-(carboxyethyl)lysine
CML: N^ε^-(carboxymethyl)lysine
CODAM: Cohort on Diabetes and Atherosclerosis Maastricht
DAF: Decay accelerating factor
3-DG: 3-Deoxyglucosone
FDR: False discovery rate
GLO1: Glyoxalase 1
GO: Glyoxal
IGM: Impaired glucose metabolism
MAC: Membrane attack complex
MG-H1: N^δ^-(5-hydro-5-methyl-4-imidazolon-2-yl)-ornithine
MGO: Methylglyoxal
NGM: Normal glucose metabolism
RAGE: Receptor for AGEs
sC5b-9: Soluble C5b-9
