# Supplementary material for: Associations of dicarbonyl stress with complement activation: the CODAM study
Source: Diabetologia. 2020 Jan 28;63(5):1032–42. doi: 10.1007/s00125-020-05098-4 (PMC7145776; doi:10.1007/s00125-020-05098-4)
Supplement: Supplementary file 1 — (PDF 391 kb) [file 125_2020_5098_MOESM1_ESM.pdf]

Electronic supplementary material (ESM):

Associations of dicarbonyl stress with complement activation: the CODAM study

The PDF file includes:

ESM Table 1 Associations of plasma α-dicarbonyls, free and protein-bound AGEs with C3a and sC5b-9 — Additional adjustment for plasma lipids, renal function, C3 levels, and glucose metabolism status

ESM Table 2 Stratified analyses for the associations of plasma α-dicarbonyls, free and protein-bound AGEs with C3a and sC5b-9 according to the presence of diabetes

ESM Table 3 Sensitivity analyses for the associations of plasma α-dicarbonyls, free and protein-bound AGEs with C3a and sC5b-9

ESM Table 4 Associations of *GLO1* polymorphism with plasma α-dicarbonyls, free and protein-bound AGEs

ESM Table 1 Associations of plasma α-dicarbonyls, free and protein-bound AGEs with C3a and sC5b-9 — Additional adjustment for plasma lipids, renal function, C3 levels, and glucose metabolism status (*n=530*)

Variable	Full model plus:	C3a (SD) β	C3a (SD) 95% CI	C3a (SD) *p* value	sC5b-9 (SD) β	sC5b-9 (SD) 95% CI	sC5b-9 (SD) *p* value
α-dicarbonyls							
**MGO (SD)**	Lipids	0.02	(−0.07, 0.10)	0.735	0.02	(−0.07, 0.11)	0.607
	GMS	0.01	(−0.08, 0.10)	0.803	0.01	(−0.09, 0.10)	0.902
	eGFR	0.02	(−0.07, 0.10)	0.719	0.01	(−0.08, 0.10)	0.851
	C3	0.00	(−0.08, 0.09)	0.940	0.01	(−0.08, 0.10)	0.826
**GO (SD)**	Lipids	−0.05	(−0.15, 0.04)	0.232	−0.11	(−0.21, −0.02)	0.019
	GMS	−0.06	(−0.15, 0.03)	0.213	−0.12	(−0.21, −0.03)	0.011
	eGFR	−0.06	(−0.14, 0.03)	0.227	−0.12	(−0.21, −0.03)	0.012
	C3	0.00	(−0.09, 0.09)	0.967	−0.08	(−0.17, 0.01)	0.086
**3-DG (SD)**	Lipids	0.01	(−0.10, 0.11)	0.885	0.09	(−0.02, 0.20)	0.100
	GMS	−0.00	(−0.12, 0.12)	0.972	0.06	(−0.06, 0.18)	0.326
	eGFR	0.00	(−0.10, 0.11)	0.981	0.08	(−0.03, 0.19)	0.162
	C3	−0.02	(−0.12, 0.08)	0.708	0.06	(−0.05, 0.17)	0.269
Free AGEs							
**CML (SD)**	Lipids	0.01	(−0.08, 0.10)	0.860	−0.03	(−0.12, 0.07)	0.576
	GMS	0.01	(−0.08, 0.10)	0.808	−0.02	(−0.11, 0.07)	0.658
	eGFR	0.02	(−0.07, 0.11)	0.678	−0.04	(−0.13, 0.06)	0.450
	C3	0.01	(−0.07, 0.10)	0.803	−0.02	(−0.11, 0.07)	0.662
**CEL (SD)**	Lipids	−0.05	(−0.14, 0.04)	0.264	−0.07	(−0.16, 0.02)	0.140
	GMS	−0.05	(−0.13, 0.04)	0.278	−0.07	(−0.16, 0.02)	0.125
	eGFR	−0.04	(−0.13, 0.05)	0.362	−0.09	(−0.18, 0.00)	0.062
	C3	−0.05	(−0.14, 0.03)	0.229	−0.07	(−0.16, 0.02)	0.121
**MG−H1 (SD)**	Lipids	−0.05	(−0.14, 0.04)	0.272	−0.04	(−0.13, 0.06)	0.431
	GMS	−0.04	(−0.13, 0.05)	0.341	−0.03	(−0.12, 0.06)	0.496
	eGFR	−0.04	(−0.13, 0.05)	0.405	−0.05	(−0.14, 0.05)	0.339
	C3	−0.04	(−0.12, 0.05)	0.377	−0.03	(−0.12, 0.06)	0.549
Protein-bound AGEs							
**CML (SD)**	Lipids	−0.09	(−0.18, 0.01)	0.070	−0.06	(−015, 0.04)	0.245
	GMS	−0.08	(−0.17, 0.01)	0.097	−0.04	(−0.14, 0.06)	0.410
	eGFR	−0.07	(−0.17, 0.02)	0.114	−0.06	(−0.15, 0.04)	0.246
	C3	−0.04	(−0.13, 0.05)	0.364	−0.02	(−0.12, 0.07)	0.662
**CEL (SD)**	Lipids	−0.16	(−0.25, −0.08)	<0.001	−0.07	(−0.16, 0.01)	0.100
	GMS	−0.17	(−0.25, −0.08)	<0.001	−0.08	(−0.17, 0.01)	0.069
	eGFR	−0.16	(−0.25, −0.08)	<0.001	−0.09	(−0.17, −0.00)	0.048
	C3	−0.17	(−0.25, −0.09)	<0.001	−0.09	(−0.17, −0.00)	0.043
**Pentosidine (SD)**	Lipids	−0.06	(−0.15, 0.03)	0.217	0.14	(0.05, 0.23)	0.003
	GMS	−0.05	(−0.14, 0.04)	0.264	0.15	(0.05, 0.24)	0.002
	eGFR	−0.05	(−0.14, 0.05)	0.314	0.14	(0.05, 0.23)	0.003
	C3	−0.03	(−0.12, 0.06)	0.534	0.16	(0.07, 0.25)	<0.001

Concentrations of α-dicarbonyls, AGEs, C3a, and sC5b-9 were standardized. Concentrations of AGEs, C3a, and triacylglycerol were log^2-transformed prior to standardization

Lipids: adjusted for age, sex, lifestyle (smoking status, alcohol consumption, physical activity, and energy intake), medication use (glucose-lowering, lipid-modifying, and/or antihypertensive), and waist circumference (full model) + plasma lipids (concentrations of triacylglycerol, total cholesterol, and HDL-cholesterol). GMS: full model + glucose metabolism status (GMS). eGFR: full model + renal function (eGFR). C3: full model + C3 concentration

ESM Table 2 Stratified analyses for the associations of plasma α-dicarbonyls, free and protein-bound AGEs with C3a and sC5b-9 according to the presence of diabetes (*n=530*)

**Variable**		**C3a (SD)**			**sC5b-9 (SD)**		
		**β**	**95% CI**	***p* value**	**β**	**95% CI**	***p* value**
α-dicarbonyls							
**MGO (SD)**	NDM	−0.04	(−0.15, 0.07)	0.487	−0.05	(−0.15, 0.06)	0.393
	T2DM	0.13	(−0.01, 0.28)	0.073	0.13	(−0.05, 0.31)	0.155
			p for interaction	0.083		*p for interaction*	0.098
**GO (SD)**	NDM	−0.10	(−0.23, 0.04)	0.154	−0.15	(−028, −0.02)	0.024
	T2DM	−0.04	(−0.15, 0.08)	0.537	−0.10	(−0.24, 0.04)	0.171
			p for interaction	0.598		*p for interaction*	0.602
**3-DG (SD)**	NDM	−0.06	(−0.24, 0.12)	0.485	−0.03	(−0.20, 0.15)	0.774
	T2DM	0.07	(−0.08, 0.21)	0.371	0.12	(−0.06, 0.30)	0.193
			p for interaction	0.168		*p for interaction*	0.248
Free AGEs							
**CML (SD)**	NDM	0.03	(−0.08, 0.24)	0.637	0.04	(−0.06, 0.14)	0.413
	T2DM	−0.05	(−0.21, 0.12)	0.567	−0.21	(−0.41, −0.01)	0.040
			p for interaction	0.535		*p for interaction*	0.010
**CEL (SD)**	NDM	−0.04	(−0.15, 0.07)	0.455	0.01	(−0.09, 0.11)	0.872
	T2DM	−0.10	(−0.25, 0.05)	0.188	−0.29	(−0.47, −0.10)	0.003
			p for interaction	0.719		*p for interaction*	0.004
**MG-H1 (SD)**	NDM	−0.03	(−0.14, 0.08)	0.581	−0.02	(−0.13, 0.09)	0.687
	T2DM	−0.09	(−023, 0.06)	0.252	−0.05	(−0.23, 0.14)	0.616
			p for interaction	0.588		*p for interaction*	0.578
Protein-bound AGEs							
**CML (SD)**	NDM	−0.09	(−0.21, 0.03)	0.143	0.05	(−0.07, 0.016)	0.410
	T2DM	−0.06	(−0.21, 0.08)	0.393	−0.24	(−0.42, −0.06)	0.010
			p for interaction	0.743		*p for interaction*	0.027
**CEL (SD)**	NDM	*−0.23*	*(−0.33, −0.12)*	<0.001	−0.02	(−0.12, 0.08)	0.703
	T2DM	−0.05	(−0.19, 0.08)	0.429	−0.21	(−0.38, −0.05)	0.013
			p for interaction	0.039		*p for interaction*	0.066
**Pentosidine (SD)**	NDM	−0.02	(−0.14, 0.09)	0.717	0.17	(0.06, 0.28)	0.003
	T2DM	−0.13	(−0.27, 0.01)	0.070	0.11	(−0.07, 0.29)	0.214
			p for interaction	0.146		*p for interaction*	0.555

Concentrations of α-dicarbonyls, AGEs, C3a, and sC5b-9 were standardized. Concentrations of AGEs and C3a were log_2-transformed prior to standardization

NDM, analyses were performed in individuals without diabetes (n=392). T2DM, analyses were performed in individuals with T2DM (n=138)

All adjusted for age, sex, lifestyle (smoking status, alcohol consumption, physical activity, and energy intake), medication use (glucose-lowering, lipid-modifying, and/or antihypertensive), and waist circumference

T2DM, type 2 diabetes mellitus

ESM Table 3 Sensitivity analyses for the associations of plasma α-dicarbonyls, free and protein-bound AGEs with C3a and sC5b-9

Variable	*Outcome*					
	C3a (SD)			sC5b-9 (SD)		
	β	95 % CI	*p* value	β	95 % CI	*p* value
	No chronic or acute infections; n=494 [a]					
α-dicarbonyls						
MGO (SD)	0.01	(−0.08, 0.10)	0.883	−0.02	(−0.11, 0.08)	0.740
GO (SD)	−0.05	(−0.14, 0.05)	0.327	−0.08	(−0.18, 0.01)	0.087
3-DG (SD)	−0.01	(−0.13, 0.10)	0.831	0.03	(−0.09, 0.14)	0.670
Free AGEs						
CML (SD)	0.03	(−0.06, 0.12)	0.518	−0.02	(−0.11, 0.08)	0.727
CEL (SD)	−0.04	(−0.13, 0.05)	0.383	−0.06	(−0.15, 0.04)	0.245
MG-H1 (SD)	−0.04	(−0.13, 0.05)	0.399	−0.02	(−0.11, 0.08)	0.737
**Protein-bound AGEs**						
CML (SD)	−0.08	(−0.18, 0.01)	0.088	−0.05	(−0.15, 0.05)	0.316
CEL (SD)	−0.18	(−0.26, −0.09)	<0.001	−0.09	(−0.18, 0.00)	0.042
Pentosidine (SD)	−0.05	(−0.14, 0.04)	0.300	0.14	(0.04, 0.23)	0.006
	No autoimmune disease; n=459 [b]					
α-dicarbonyls						
MGO (SD)	−0.01	(−0.1, 0.08)	0.805	0.01	(−0.09, 0.11)	0.832
GO (SD)	−0.06	(−0.15, 0.04)	0.253	−0.11	(−0.20, −0.01)	0.038
3-DG (SD)	−0.01	(−0.12, 0.10)	0.836	0.06	(−0.05, 0.18)	0.280
Free AGEs						
CML (SD)	0.00	(−0.09, 0.10)	0.933	−0.03	(−0.12, 0.07)	0.572
CEL (SD)	−0.07	(−0.16, 0.03)	0.158	−0.08	(−0.18, 0.01)	0.096
MG-H1 (SD)	−0.05	(−0.14, 0.05)	0.329	−0.05	(−0.15, 0.04)	0.285
**Protein-bound AGEs**						
CML (SD)	−0.11	(−0.21, −0.01)	0.029	−0.06	(−0.16, 0.04)	0.263
CEL (SD)	−0.20	(−0.28, −0.11)	0.000	−0.09	(−0.18, 0.00)	0.054
Pentosidine (SD)	−0.05	(−0.15, 0.04)	0.284	0.14	(0.04, 0.24)	0.006
	No malignant disease and/or cancer; n=508 [c]					
α-dicarbonyls						
MGO (SD)	0.01	(−0.07, 0.10)	0.741	0.03	(−0.06, 0.12)	0.568
GO (SD)	−0.06	(−0.15, 0.03)	0.168	−0.11	(−0.20, −0.02)	0.018
3-DG (SD)	0.01	(−0.10, 0.12)	0.846	0.09	(−0.02, 0.20)	0.111
Free AGEs						
CML (SD)	0.00	(−0.09, 0.09)	0.969	−0.02	(−0.11, 0.07)	0.701
CEL (SD)	−0.06	(−0.15, 0.03)	0.200	−0.07	(−0.16, 0.02)	0.114
MG-H1 (SD)	−0.05	(−0.14, 0.04)	0.273	−0.03	(−0.12, 0.06)	0.524
**Protein-bound AGEs**						
CML (SD)	−0.08	(−0.17, 0.01)	0.096	−0.06	(−0.16, 0.03)	0.213
CEL (SD)	−0.17	(−0.25, −0.08)	<0.001	−0.09	(−0.17, 0.00)	0.051
Pentosidine (SD)	−0.06	(−0.15, 0.03)	0.201	0.14	(0.05, 0.24)	0.002
	No liver disease; n=524 [d]					
α-dicarbonyls						

**MGO (SD)**	0.01	(−0.07, 0.10)	0.749	0.02	(−0.07, 0.11)	0.602
**GO (SD)**	−0.05	(−0.14, 0.04)	0.273	−0.11	(−0.20, −0.02)	0.018
**3-DG (SD)**	0.01	(−0.10, 0.11)	0.925	0.07	(−0.04, 0.18)	0.194
Free AGEs						
**CML (SD)**	0.01	(−0.08, 0.10)	0.834	−0.02	(−0.11, 0.07)	0.712
**CEL (SD)**	−0.05	(−0.13, 0.04)	0.286	−0.06	(−0.15, 0.03)	0.163
**MG-H1 (SD)**	−0.05	(−0.14, 0.04)	0.298	−0.03	(−0.12, 0.06)	0.573
**Protein-bound AGEs**						
**CML (SD)**	−0.08	(−0.17, 0.01)	0.074	−0.05	(−0.14, 0.05)	0.327
**CEL (SD)**	−0.17	(−0.25, −0.08)	0.000	−0.08	(−0.16, 0.01)	0.073
**Pentosidine (SD)**	−0.05	(−0.14, 0.04)	0.278	0.15	(0.05, 0.24)	0.002

Concentrations of α-dicarbonyls, AGEs, C3a, and sC5b-9 were standardized. Concentrations of AGEs and C3a were log_2-transformed prior to standardization

All adjusted for age, sex, lifestyle (smoking status, alcohol consumption, physical activity, and energy intake), medication use (glucose-lowering, lipid-modifying, and/or antihypertensive), and waist circumference

[a] 36 participants with acute or chronic infections were excluded. [b] 71 participants were excluded, of which 61 with a (suspected) history of autoimmune disease and 10 missing information on autoimmune disease. [c] 22 participants were excluded, of which 18 with a self-reported current malignant condition/cancer and 4 missing information on malignant disease and/or cancer. [d] 6 participants with a self-reported current liver disease were excluded

ESM Table 4 Associations of *GLO1* polymorphism with plasma α-dicarbonyls, free and protein-bound AGEs (*n=504*)

		rs2736654					rs1049346				
		TT (reference)	GT		GG		GG (reference)	AG		AA	
	Model		β	*p* value	β	*p* value		β	*p* value	β	*p* value
**Outcomes**											
**MGO (SD)**	1	–	−0.18	0.074	−0.19	0.156	–	0.09	0.446	0.02	0.861
	2	–	−0.20	0.042	−0.16	0.234	–	0.09	0.411	0.08	0.526
	3	–	−0.21	0.036	−0.15	0.258	–	0.09	0.410	0.08	0.508
Free AGEs											
**CML (SD)**	1	–	−0.04	0.717	−0.21	0.099	–	−0.10	0.340	−0.15	0.232
	2	–	−0.04	0.724	−0.18	0.153	–	−0.14	0.197	−0.20	0.117
	3	–	−0.04	0.708	−0.18	0.160	–	−0.14	0.197	−0.19	0.120
**CEL (SD)**	1	–	−0.10	0.326	−0.12	0.375	–	−0.11	0.317	0.09	0.478
	2	–	−0.10	0.298	−0.09	0.490	–	−0.14	0.211	0.09	0.497
	3	–	−0.11	0.271	−0.08	0.529	–	−0.14	0.212	0.09	0.478
**MG-H1 (SD)**	1	–	−0.03	0.776	−0.01	0.961	–	−0.08	0.443	0.02	0.845
	2	–	−0.02	0.846	0.05	0.702	–	−0.14	0.199	−0.04	0.770
	3	–	−0.01	0.885	0.04	0.739	–	−0.14	0.198	−0.04	0.751
Protein-bound AGEs											
**CML (SD)**	1	–	−0.06	0.576	−0.03	0.796	–	−0.05	0.625	0.08	0.513
	2	–	−0.03	0.799	−0.07	0.615	–	−0.07	0.548	0.05	0.704
	3	–	0.01	0.908	−0.11	0.355	–	−0.07	0.511	0.03	0.824
**CEL (SD)**	1	–	−0.16	0.121	−0.00	0.990	–	0.02	0.831	0.05	0.697
	2	–	−0.15	0.142	−0.01	0.957	–	−0.01	0.962	0.01	0.931
	3	–	−0.15	0.140	−0.01	0.964	–	−0.01	0.962	0.01	0.930
**Pentosidine (SD)**	1	–	−0.03	0.757	−0.11	0.401	–	−0.08	0.491	−0.12	0.360
	2	–	−0.04	0.678	−0.14	0.283	–	−0.06	0.559	−0.08	0.537
	3	–	−0.01	0.924	−0.18	0.141	–	−0.07	0.528	−0.10	0.417

The reference categories are the genotypes that were reported to be associated with the highest GLO1 activity (see reference 22–24 in the main text)

Concentrations of MGO and AGEs were standardized. Concentrations of AGEs were log_2-transformed prior to standardization

Model 1: adjusted for age and sex. Model 2: model 1 + lifestyle (smoking status, alcohol consumption, physical activity, and energy intake) and medication use (glucose-lowering, lipid-modifying, and/or antihypertensive). Model 3: model 2 + waist circumference
